# Surface Modification of Magnetic Nanoparticles by Carbon-Coating Can Increase Its Biosafety: Evidences from Biochemical and Neurobehavioral Tests in Zebrafish

**DOI:** 10.3390/molecules25092256

**Published:** 2020-05-11

**Authors:** Nemi Malhotra, Gilbert Audira, Jung-Ren Chen, Petrus Siregar, Hua-Shu Hsu, Jiann-Shing Lee, Tzong-Rong Ger, Chung-Der Hsiao

**Affiliations:** 1 Department of Biomedical Engineering, Chung Yuan Christian University, Chung-Li 32023, Taiwan; nemi.malhotra@gmail.com; 2 Department of Chemistry, Chung Yuan Christian University, Chung-Li 32023, Taiwan; gilbertaudira@yahoo.com; 3 Department of Bioscience Technology, Chung Yuan Christian University, Chung-Li 32023, Taiwan; siregar.petrus27@gmail.com; 4 Department of Biological Science & Technology, College of Medicine, I-Shou University, Kaohsiung 82445, Taiwan; jrchen@isu.edu.tw; 5 Department of Applied Physics, National Pingtung University, Pingtung 90007, Taiwan; hshsu@mail.nptu.edu.tw; 6 Center for Nanotechnology, Chung Yuan Christian University, Chung-Li 32023, Taiwan

**Keywords:** carbon magnetic nanoparticles, zebrafish, behavior analysis, neurotransmitters, ecotoxicity, phenomic analysis

## Abstract

Recently, magnetic nanoparticles (MNPs) have gained much attention in the field of biomedical engineering for therapeutic as well as diagnostic purposes. Carbon magnetic nanoparticles (C-MNPs) are a class of MNPs categorized as organic nanoparticles. C-MNPs have been under considerable interest in studying in various applications such as magnetic resonance imaging, photothermal therapy, and intracellular transportof drugs. Research work is still largely in progress for testing the efficacy of C-MNPs on the theranostics platform in cellular studies and animal models. In this study, we evaluated the neurobehavioral toxicity parameters on the adult zebrafish (*Danio rerio*) at either low (1 ppm) or high (10 ppm) concentration level of C-MNPs over a period of two weeks by waterborne exposure. The physical properties of the synthesized C-MNPs were characterized by transmission electron microscopy, Raman, and XRD spectrum characterization. Multiple behavior tests for the novel tank, mirror biting, predator avoidance, conspecific social interaction, shoaling, and analysis of biochemical markers were also conducted to elucidate the corresponding mechanism. Our data demonstrate the waterborne exposure of C-MNPs is less toxic than the uncoated MNPs since neither low nor high concentration C-MNPs elicit toxicity response in behavioral and biochemical tests in adult zebrafish. The approach combining biochemical and neurobehavioral approaches would be helpful for understanding C-MNPs association affecting the bioavailability, biosafety, interaction, and uptake of these C-MNPs in the living organism.

## 1. Introduction

The unique characteristics of magnetic nanoparticles (MNPs) have demonstrated great potential in various fields of biomedical engineering applications such as bioseparation, contrast enhancement for magnetic resonance imaging, and intracellular drug delivery [1,2,3,4,5,6]. The uncoated MNPs exhibit various beneficial qualities of high surface to volume ratio and high magnetic moment, permitting manipulation of these MNPs by an external magnetic field [7,8]. However, uncoated MNPs are not stable which might lead to uncontrolled magnetic behavior, loss of magnetism, lack of dispersibility, aggregation. and easy oxidization [9,10,11]. Due to small size and comparable dimensions with cellular components, protein molecules, and viruses, MNPs afford the potentiality of interaction with the fundamental biological processes [12]. The small size of these nanoparticles is essential to evade the immune system, for efficient circulation and life-span inside the body [13]. Fe_3_O_4_ MNPs have been studied extensively as an important material of choice for synthetization of MNPs owing to its properties of ease of synthetization, functionality, superparamagnetism, and low toxicity. However, Fe_3_O_4_ in a small size (5–15 nm) has been shown to demonstrate chemical corrosion instability, which may cause partial oxidation into γ-Fe_2_O_3_ [14,15], and also aggregate under the influence of van der Waal forces, due to high surface energy among these particles [16].

Because the quality and surface chemistry play important roles in biomedical applications, a variety of polymers have been developed for coating in the biomedical sector, for improvement in the performance of useful applications advancement, for example, polymers poly-d, l-lactic-co-glycolic acid (PLGA), polyethylene glycol (PEG), polyethylene oxide (PEO), poly-*N*-isopropylacrylamide (PNIPA), polyethyleneimine (PEI), polyacrylic acid (PAA), polyvinyl alcohol (PVA) [17] and noble materials (gold, silver) [14]. Therefore, various strategies have been devised to make Fe_3_O_4_ MNPs more effective and useful, i.e., organic and inorganic coating including surfactants, polymers [18,19], silica [20], and carbon [11,21,22], have been used for coating of these nanoparticles surface to make them corrosion resistant and more biocompatible.

A carbon shell coating is usually formed through a high-temperature strengthening process, which carbonizes hydrocarbon (HC) precursors but also reduces iron oxide [14,23,24]. It is known that the morphology and internal structure of these MNPs are significant to comprehend the concluding properties of the particle. Carbon is an outstanding material because of its biocompatibility and its stability over the entire pH range and ease of further surface modifications, and carbon-coated magnetic nanoparticles demonstrate extensive stability in alkaline or acid medium, elevated biocompatibility, low toxicity, and good saturation magnetization [11,25,26,27].

Available in vitro and in vivo toxicity studies are scarce in the field of uncoated or coated MNPs, although the toxicity of different types of carbon nanoparticles, i.e., fullerenes [28,29,30,31], carbon nanotubes (single wall and multiple walls) [32,33,34,35], carbon dots [36,37,38], carbon MNPs [23,39] and graphenes [40,41] have been demonstrated, in various cell lines and animals. In previous work, we have tested the chronic toxicity of the uncoated Fe_3_O_4_ MNPs in adult zebrafish and demonstrated long term waterborne MNPs exposure can reduce locomotion activity and aggressiveness [42]. To the best of our knowledge, the potential toxicity of carbon surface-modified MNPs hasnot yet beenwell tested in living organisms, like zebrafish. The reported studies have described toxicity to be dependent on the type of coating, exposure time, and dose [43,44], whereas, other studies have suggested toxicity to be dependent on the shape, size and surface charge which in turn affect the cell cycle, apoptosis and endocytic pathways [45,46,47,48]. In a previous study, synthesized multi-walled carbon nanotubes with average diameter ~500 nm did not present cytogenotoxicity in zebrafish aquatic model, with minimal disturbances in gills after 72 h exposure [35]. The carbo-iron nanomaterial made for remediation of contaminated aquifiers showed no effect on survival and growth of different life stages of zebrafish in 48 h, 96 h, and semi-static 34 days study with particle diameters 266–497 nm with natural elimination from the fish gut [49]. In another study, starch coated MNPs when exposed to zebrafish in bare and coated form induced inflammation and oxidative stressas analyzed by RNA-sequence, also bare MNPs caused more cytotoxicity to gills and coated MNPs trigger more harm to the liver. Overall, suggesting that the toxicity of MNPs is based on tissue and particle surface chemistry [43]. It is still an unsettled issue to assess the toxicity of MNPs, either coated or uncoated. Particularly, in the case of carbon-coated MNPs, it is still a long way to go, either in vitro or in vivo, for analyzing the toxicity of particles in terms of different parameters. In our previous work, we have reported thatroom-temperature ferrimagnetic enhancement can be achieved by using a controlled amount of starch precursor for the formation of carbon-coated Fe_3_O_4_ MNPs hydrothermally. During the hydrothermal process, the preparation temperature was found to be crucial for the carbonization of starch precursor and for magnetic enhancement [24]. The physical property of C-MNPs wascharacterized by transmission electron microscopy (TEM), Raman spectroscopy, fourier transform infra red spectroscopy (FTIR), x-ray powder diffraction (XRD) spectrum and magnetization hysteresis loop. The toxicity and stress response of C-MNPs to adult zebrafish were explored at both biochemical and behavioral levels. According to the best of our knowledge, we are the first group to present a study based on toxic effects of carbon-coated MNPs on the adult zebrafish on comprehensive behavioral and biochemical levels.

## 2. Material and Methods

### 2.1. C-MNPs Synthesis and Characterization

Iron oxide magnetic nanoparticles were synthesized using the co-precipitation method, using FeCl_3_6H_2_O and FeSO_4_7H_2_O at a 1:1 molar ratio. NH_4_OH was slowly added to the aqueous mixture until pH was equal to 10 at room temperature. Once the black iron oxide particles were obtained, they were separated from the liquid by using a bar magnet and washed with absolute ethanol thrice to attain final particles. The resultant iron oxide nanoparticles were vacuum dried at room temperature. After obtaining the iron oxide nanoparticles, 0.5 g Fe_3_O_4_ nanoparticles were dispersed in deionized water with soluble 0.19 g of starch precursor for the formation of Fe_3_O_4_@C nanoparticles through the hydrothermal method [24]. These dispersion mixtures were then placed in an autoclave and maintain at 200 °C for 12 h. The resulting Fe_3_O_4_@C NPs were collected after the completion of this reaction. Fe_3_O_4_@C NPs were washed with deionized water, then with absolute ethanol and vacuum dried. The Fe_3_O_4_@C NPs thus obtained were characterized by a transmission electron microscopy (TEM, JEOL JEM-2100F/Cs STEM) to inspect the shape, size, and dispersion of Fe_3_O_4_@C NPs. In addition, a vibrating sample magnetometer (VSM, Lake Shore 7400 system) was used to observe saturation magnetization and M-H loop measurements under the magnetic field (H) up to 15,000× g. Thermo-gravimetric analysis (TGA) of all samples were done using SDT Q600 instrument from TA instrument. The temperature ranged from 25 to 300 °C and the heating rate of TGA curves was 10 °C/minin a nitrogen atmosphere for the evaluation of the mass fraction of the particles.

### 2.2. XRD, Raman and FTIR Spectrum

X-ray diffraction (XRD) was performed at beamline BL01C, National Synchrotron Radiation Research Center (NSRRC, Hsinchu, Taiwan). A monochromatic X-ray beam with a wavelength of 0.6888 Å was used as the probing source. Raman measurements were performed using a microscopic Raman system (RAMaker, Protrustech Co., Ltd., Taiwan). An exciting line of 532 nm was supplied by a diode laser (CNI). For the MNPs, which were more prone to oxidation by laser heating, the output power of the Raman excitation source was reduced to 1 mW. The exposure time was 60 s with two accumulations. The instrument calibration was done using a silicon standard where the band is generally observed at 520 cm^−1^. The corresponding spectral resolution was in the range of 1 cm^−1^. A Jasco FTIR-6700 spectrometer was used in transmission mode for obtaining the IR spectra of the Fe_3_O_4_ and Fe_3_O_4_@C particles. The wavenumber range was 400–4000 cm^−1^, and the spectra were obtained at room temperature with 0.25 cm^−1^ resolution.

### 2.3. Zebrafish Ethics and Husbandry

Zebrafish (*Danio rerio*) were care of according to the approved procedures and protocols by the Chung Yuan Christian University (Number: CYCU109001, issue date 20 Jan. 2020). All the procedures performed on animals were according to the guidelines. Adult wild type AB strain zebrafish (*Danio rerio*), aged around 6 to 7 months, with no sexual preference, were maintained in a recirculating aquatic system at 27.8 °C with a 10/14 h dark/light cycle. Reverse osmosis (pH 7.0–7.5) was used to filter the circulating water in the aquarium. To ensure zebrafish uptake of C-MNPs, zebrafish were fed with fresh *Artemia* only once every two days.

### 2.4. Zebrafish Exposure to Magnetic Nanoparticles

Adult zebrafish in good physical shape were segregated into three different 50 L water tanks, containing 20 L of fish water. Each tank contained 20 zebrafish. We followed the principle of 3R (replacement, reduction, and refinement) to diminish the sacrifice of adult zebrafish [50]. The adult zebrafish were incubated with C-MNPs at 0 ppm as control, 1 ppm as a low dose, and 10 ppm as high dose, respectively, for performing the following behavioral and biochemical tests. C-MNPs were weighed and diluted in to double distilled water for sonication in an ultrasonicator, before their addition in the fish tanks. The fish tanks were cleaned and fish water was changed every two days, with the change of new C-MNPs and fish feed to avoid infection due to poor water quality. After the predetermined exposure time, behavioral tests (novel tank, mirror biting, shoaling, predator avoidance, and social interaction tests) were performed with the zebrafishes in all three groups. Results were compared between the control and treated groups. All the important behavioral endpoints measured in this study and their definition are summarized in Appendix A Table A1.

### 2.5. Novel Tank Test

The ability of the zebrafish to adapt to a new environment was evaluated by the novel tank test. For the test, a single fish was introduced into a test trapezoid tank, with dimensions: 22 cm bottom, 28 cm top, 15.2 cm high, and 15.9 cm along the diagonal side, filled with ~1.25 L of fish water, as described in the previous [51]. For novel tank tests, video recording started immediately after fish immersion into the test tanks with 1 min of recording every 5 min for 30 min. The novel tank test was later analyzed on six different endpoints of average speed, freezing time movement ratio, time in top duration, number of entries to the top, latency to enter the top, and total distance traveled in the top [52].

### 2.6. Aggressiveness Test

The mirror biting test is devised to evaluate the aggressiveness in the zebrafish [37,38,39]. The tank with the same dimensions as described above was filled with ~1.25 L of fish water and a mirror was placed at one side of the tank. Later, zebrafish were transferred into the tank and allowed to acclimate for 1 min. Henceforth, the zebrafish aggressive behavior was recorded for 5 min, according to our previous protocol and six significant endpoints of average speed, mirror biting time percentage, longest duration in the mirror side, freezing time movement ratio, swimming time movement ratio, and rapid movement time ratio were recorded, measured, and analyzed [52].

### 2.7. Predator Avoidance Test

Predator avoidance test was carried out to assess the reaction of zebrafish to both visual and olfactory cues when confronted with the predator fish. This test help analyze the changes in the aggressiveness of zebrafish. The same-sized tanks as mentioned above were each filled with ~1.25 L of fish water and segmented into two halves by a transparent separator [52]. We put two convict cichlid *(Amatitlanianigrofasciata)*, as the predator, into one side of the tank with zebrafish to be tested on the other side. Initial acclimation was allowed for both predator and zebrafishes for 1 min before starting the 5 min recording. Later, endpoints such as average speed, predator approaching time percentage, average distance to the separator, a freezing time movement ratio, a swimming time movement ratio, and rapid movement time ratio were measured and analyzed.

### 2.8. Shoaling Test

Zebrafish shoaling is an innate behavior that they show by swimming together in a group to avoid being captured by predators and reducing anxiety levels. The shoaling test was conducted to assess the shoaling formation ability of zebrafish. The same-sized tanks as described above were each filled with ~1.25 L of fish water. Fish in a group of three were introduced into each water tank. After allowing the fish to acclimate for 1 min, a 5 min recording was started according to our previous protocol and six endpoints, namely, average speed, time in the top duration, average shoal area, average inter-fish distance, average nearest neighbor distance, and average furthest neighbor distance were measured and analyzed [52].

### 2.9. Social Interaction Test

In order to assess the ability of zebrafish to interact with their conspecifics, a social interaction test was devised. A transparent glass separator dividing the tank into two halves was used in the same sized tank filled with ~1.25 L fish water according to the published protocol [52]. The fish to be analyzed was added on one side of the tank with their conspecific into the other side of the tank. After the initial acclimation of 1 min, a 5 min video recording was taken and six different endpoints of interaction time percentage, longest duration in the separator side, average speed, and average distance to the separator were observed and calculated [52].

### 2.10. Tissue Preparation and Total Protein Determination

After the completion of all the behavioral analyses, zebrafish were sacrificed by immersing them into high dose MS222 and whole brains extracted from every single zebrafish for each independent assay. Three whole zebrafish brains were used to prepare a single homogenate for each sample, standardized in volumes of 50 (*v*/*w*) of ice-cold phosphate-buffered saline (PBS) at pH 7.2, and later used bullet blender (Next Advance, Inc., Troy, NY, USA) to perform tissue homogenization. All the prepared samples were centrifuged at 4000 rpm for 20 min at 4 °C, and the supernatant was kept in micro Eppendorf tubes in the freezer at −80 °C for further investigation. Total protein analysis of the prepared fish brain sample was done using a Pierce BCA (bicinchoninic acid) protein assay kit (23225, ThermoFisher Scientific, Waltham, MA, USA). The color formation in the protein assay kit was analyzed at 562 nm using a microplate reader (Multiskan GO, Thermo Fisher Scientific, Waltham, MA, USA) according to our published method [52,53].

### 2.11. Quantification of Ferric (metal) Content, The Stress Hormone, and Oxidative Stress Markers Were Analyzed in Brain Tissues

The ferric (metal) content in the brain tissue sample, was determined using a colorimetric-based iron assay kit (A039-2, Nanjing Jiancheng Bioengineering Institute, Nanjing, China). Stress hormones of catecholamine, cortisol, and metallothionein were measured by using commercial target-specific ELISA kits (ZGB-E1575, ZGB-E1562, ZGB-E1562, Zgenebio Inc., Taipei, Taiwan). The reactive oxygen species (ROS) was performed using an ELISA kit (ZGB-E1561, Zgenebio Inc., Taipei, Taiwan), as per the manufacturer’s instructions. For analyzing the energy, adequate oxygen supply, and DNA damage evaluation, was done by measuring hypoxia-inducible factor 1-alpha (HIF1-α), adenosine-5′-Triphosphate (ATP), creatine kinase (CK), and ssDNA contents using commercial target-specific ELISA kits (ZGB-E1643, ZGB-E1580, ZGB-E1581, ZGB-E1595, Zgenebio Inc. The catalase (CAT) test to detect the presence of catalase protein in the tissue lysates of the zebrafish brain tissue samples and lipid peroxidation markers of thiobarbituric acid reactive substances (TBARS) to determine the lipid peroxidation of respective brain tissue sample was done using a commercial target-specific ELISA kit (ZGB-E1582, Zgenebio Inc.).

### 2.12. Neurotransmitters Determination in Zebrafish Brain Tissues

The prepared sample of whole-brain tissue lysates was quantitatively analyzed for several different neurotransmitters’ endeavors by using ELISA kits according to the instruction by the manufacturer. A pool of brain tissues from three individual zebrafish consisted of one sample. The tests were accomplished in triplicates using a total of nine fish per group for uniformity. Acetylcholinesterase (AChE), acetylcholine (ACh), serotonin (5-HT), dopamine (DA), GABA levels were determined using an ELISA kit (ZGB-E1637, ZGB-E1585, ZGB-E1572, ZGB-E1573, ZGB-E1574, purchased from Zgenebio Inc.), respectively, according to the instructions provided by the manufacturer. The absorbance of each of the samples was analyzed at 450 nm using a microplate reader (Multiskan GO, Thermo Fisher Scientific). The relative concentration of the target protein was extrapolated from the standard curve generated from the standard provided by the commercial kits.

### 2.13. PCA, Heatmap, and Clustering Analysis

All of the endpoint’s value differences between control and treated fish in every behavior test were calculated and input in to excel file using Microsoft Excel. All of the important behavioral endpoints were listed in Supplementary Table S1 while their descriptions in each test were discussed in the previous study [51]. Next, the excel file was converted to a comma-separated values type file (.csv) and uploaded to ClustVis (https://biit.cs.ut.ee/clustvis/), a web tool designed for visualizing and clustering multivariate data. Since the data range covered multiple magnitudes and the smallest value on the data is 0, data transformation by ln(x + 1) was performed during the pre-processing step. After data transformation, several endpoints (1–1, 1–4, 1–6) were removed since they had constant values. Later, unit variance scaling for each row was carried out in order to treat each variable equally and SVD with imputation method was used to calculate principal components as there were no missing values in the dataset [54]. After the data was processed, PCA and heatmap results were exported and saved in the computer system.

### 2.14. Statistical Analysis

All the statistical analyses of the results were plotted and calculated by using GraphPad Prism (GraphPad Software version 7 Inc., La Jolla, CA, USA). Each fish group was compared to the control group, using either one-way or two-way ANOVA or Kruskal-Wallis tests and followed by Dunn’s or Dunnett’s multiple comparison test as mentioned in the figure caption. A significant difference between control and treated groups was marked as “*” if *p* < 0.05; “**” if *p* < 0.01; “***” if *p* < 0.001; and “****” if *p* < 0.0001.

## 3. Results

### 3.1. Carbon Magnetic Nanoparticles (C-MNPs) Characterization

**Figure 1:**
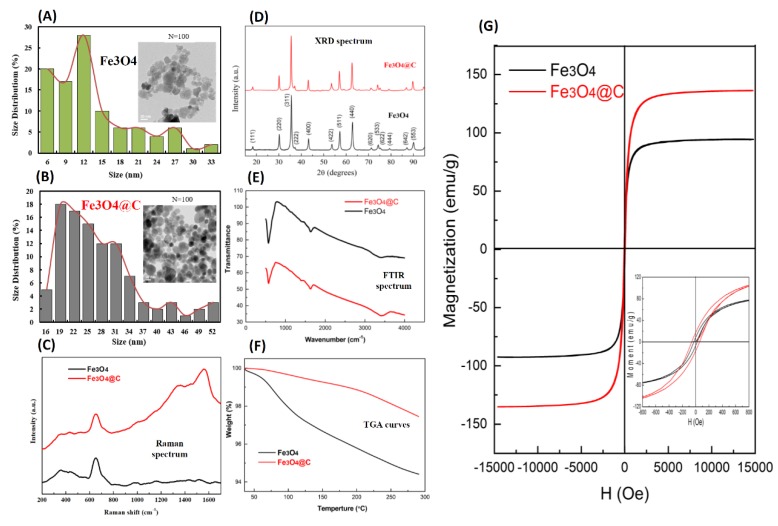
Transmission electron microscope images and size distribution of (**A**) pristine Fe_3_O_4_, magnetic nanoparticles (Fe_3_O_4_), and (**B**) carbon-coated Fe_3_O_4_ MNPs (Fe_3_O_4_@C). (**C**) Comparison of Raman spectrum between pristine Fe_3_O_4_ magnetic nanoparticles (MNPs) (black color) and carbon-coated Fe_3_O_4_ MNPs (red color). (**D**) Comparison of the XRD (X-ray Diffraction) spectrum between pristine Fe_3_O_4_ MNPs (black color) and carbon-coated Fe_3_O_4_ MNPs (red color). (**E**) Fourier-transform infrared spectroscopy (FTIR) spectra of pristine Fe_3_O_4_ and carbon-coated Fe_3_O_4_ NPs. (**F**) TGA (Thermogravimetric analysis) curves of pristine Fe_3_O_4_ and carbon-coated Fe_3_O_4_ NPs. (**G**) Magnetization versus applied field for pristine Fe_3_O_4_ and carbon-coated Fe_3_O_4_ NPs. The insert shows hysteresis loops.

A combination of co-precipitation and the hydrothermal method was used for the synthesis of uncoated Fe_3_O_4_ MNPs and carbon-coated Fe_3_O_4_ MNPs, respectively.The C-MNPs thus acquired, are spherical in shape, the dark core of Fe_3_O_4_ nanoparticles and grey carbon shell is noticeable in Figure 1B as determined by transmission electron microscopy (TEM), the image in inset shows good dispersion of resultant magnetic nanoparticles [24]. The starch precursor was used for coating Fe_3_O_4_ MNPs at 200 °C, the coating thickness was assessed to be 2 nm. In addition, we used Raman and XRD (X-ray Diffraction) spectrum to compare the physical properties between bare Fe3O4 MNPs and carbon-coated Fe3O4 MNPs. Figure 1C shows the Raman spectrum for the phase identifications of these two samples. The spectrum of the Fe3O4 MNPs had themain peak at 660–680 nm, consistent with the peaks described in the related literature. In the high wavenumber region (1100–1800 cm^−1^), the C-MNPs sample exhibited a typical spectrum of diamond-like carbon. The intensity ratio (ID/IG) was found to correlate linearly with the sp2/sp3 carbon ratio. The prominent broad band at high frequency could be deconvoluted into two different broad band at 1540–1580 cm^−1^ (G band) and at 1330–1350 cm^−1^ (D band). According to our Raman analysis, the hydrothermal carbonization process converted organic starch into a carbon-like product at 200 °C. Furthermore, these two samples exhibited similar XRD patterns (Figure 1D), suggesting the crystalline form was nearly unchanged during the hydrothermal process. All these diffraction peaks could be indexed to a spinel structure of magnetite (ICSD # 75627). The average crystallite sizes (D_311_) of the MNPs and C-MNPs were estimated to be 18 and 24 nm, respectively, through Scherrer’s formula. Figure 1E presents the FTIR (Fourier-transform infrared spectroscopy) spectra of as-prepared Fe_3_O_4_ and Fe_3_O_4_@C MNPs. The band observed at 572 cm^−1^ with a shoulder around 700 cm^−1^ is comparable with those of magnetite. The broad band around 3435 cm^−1^ and the weak one at 1634 cm^−1^ in these two samples are attributed to adsorbed water. In addition, thermogravimetric analysis (TGA) measurements for the uncoated and coated magnetite samples both exhibited the weight reduction in the 40–115 °C range (Figure 1F). In the temperature range, the Fe_3_O_4_ and Fe_3_O_4_@C had a mass drop of ~2.6% and 0.4%, respectively, caused mainly by water vaporization from the samples. The result shows that the carbon-coated Fe_3_O_4_ MNPs exhibited a hydrophobic behavior compared to the pristine Fe_3_O_4_ MNPs. The magnetization (*M*) versus field (*H*) curve at 300K for samples of synthetic magnetite is shown in Figure 1G. It was observed that synthetic Fe_3_O_4_ had a saturation magnetization (*Ms*) of 94 emu/g and coercivity of 22 Oe and showed a nearly superparamagnetic behavior. The *Ms* value and coercivity were 135 emu/g and 57 Oe for Fe_3_O_4_@C sample. An increase in magnetization value could be observed in the carbon-coated Fe_3_O_4_ MNPs.

### 3.2. Reduction in Locomotor Activity on Exposure to the Low and High Concentrations of C-MNPs

**Figure 2:**
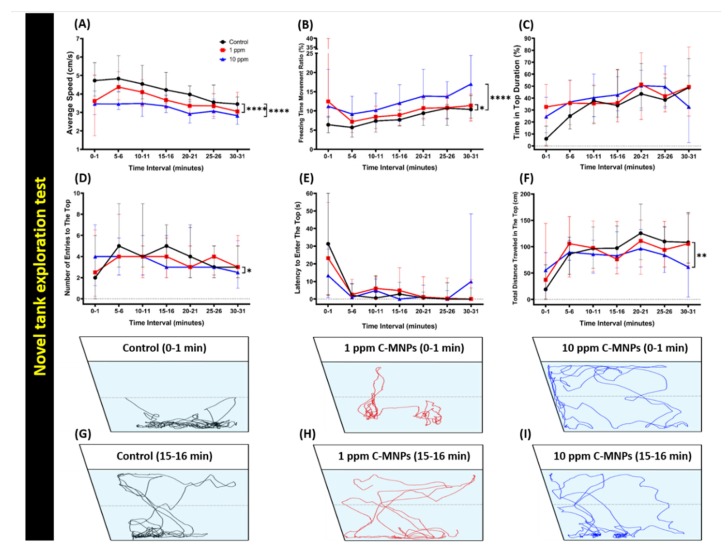
Behavior endpoints of control and carbon magnetic nanoparticles (C-MNPs)-exposed zebrafish in the novel tank test after two weeks’ exposure. (**A**) Average speed, (**B**) freezing time movement ratio, (**C**) time in the top duration, (**D**) the number of entries to the top, (**E**) latency to enter the top, and (**F**) total distance traveled in the top were analyzed. (**G**–**I**) The locomotor trajectories of control as well as 1 and 10 ppm C-MNPs-exposed fish in the novel tank test. The black line represents the control group, the red line represents the low concentration C-MNPs group (1 ppm), and the blue line represents the high concentration C-MNPs group (10 ppm). The data are expressed as the median with interquartile range and were analyzed by two-way ANOVA with Geisser-Greenhouse correction. To observe the main column (C-MNPs) effect, Dunnett’s multiple comparison test was carried out. (*n* = 20, * *p* < 0.05, ** *p* < 0.01, **** *p* < 0.001).

The novel tank test is devised to analyze the fish adaptability and exploration pattern when introduced in a new environment. It has been shown earlier, when zebrafish are introduced into a new environment they exhibit anxiety and bottom-dwelling behavior at first, but once they acclimatize to the new environment, their stress/anxiety level is reduced and they start to explore the tank, by moving towards other areas of the tank [52,55,56]. After the decided period of two weeks of incubation with C-MNPs, the fish were tested for their locomotor activity and exploratory behavior in a new tank. The locomotor activity of zebrafish revealed a reduction in the low concentration group (1 ppm) and high concentration (10 ppm) in comparison to the control group (0 ppm). This phenomenon was shown by a low level of average speed and accompanying a high level of freezing time ratio displayed by low and high concentration group which indicated locomotor activity alterations (Figure 2A,B). In addition, the decline in average speed was also accompanied by a significant level of freezing time movement ratio in the low and high concentration groups (Figure 2B). Next, an alteration in treated zebrafish exploratory behavior can be shown by the changes in time spent in the top duration, the number of entries to the top, latency to enter the top and total distance traveled at the top. In this experiment, a slight difference was observed between the low and high concentration groups in relation to zebrafish exploratory behavior. These significant changes in the number of entries to the top and total distance traveled in the top were observed in the high concentration group in comparison to the low concentration and control groups (Figure 2C,F). Taken together, the high concentration C-MNPs-exposed group presented more substantial alterations in the exploratory behavior than the low concentration C-MNPs-exposed group. The trajectories of locomotion activity for the control group, 1 ppm, and 10 ppm group C-MNPs-exposed group after 0–1 min (upper panel) and 15–16 min (bottom panel) acclimation are summarized in Figure 2G–I and Video S1 for better visualization fish movements. The result obtained here in this test suggested that exposure of C-MNPs to zebrafish at either low (1 ppm) or high (10 ppm) concentration hampered locomotor and exploratory behavior of the zebrafish.

### 3.3. C-MNPs Exposure Did Not Change Aggressive Behavior in Zebrafish

**Figure 3:**
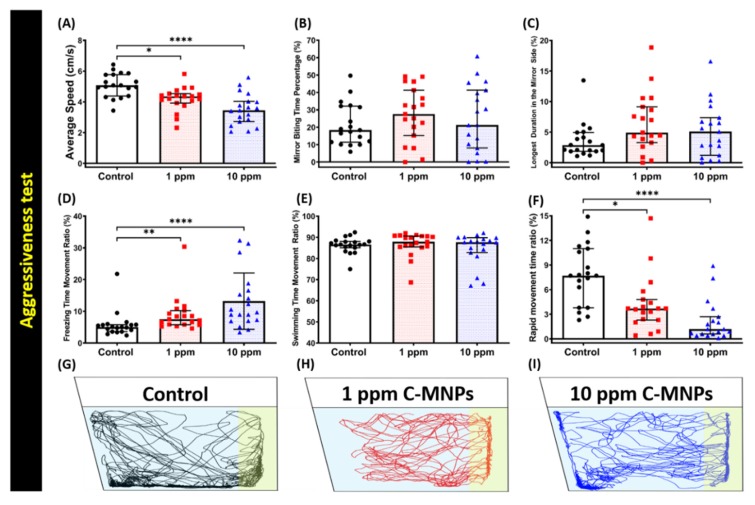
Biting behavior endpoint comparisons between the control group, 1 ppm, and 10 ppm C-MNPs-exposed zebrafish groups after two weeks of exposure. (**A**) Average speed (**B**) mirror biting time percentage (**C**) longest duration in the mirror side (**D**) freezing time movement ratio (**E**) swimming time movement ratio, and (**F**) rapid movement time ratio were analyzed. (**G**–**I**) The 5 min locomotor trajectories of control, 1 and 10 ppm C-MNPs-exposed fish in the mirror biting test. The data are expressed as the median with interquartile range and were analyzed by the Kruskal-Wallis test continued with Dunn’s multiple comparisons test as a follow-up test (*n* = 20, * *p* < 0.05, ** *p* < 0.01, **** *p* < 0.001).

The aggressiveness of zebrafishes can be monitored by analyzing the frequency at which they bite their own mirror images, which is a simple yet effective method established in the literature to test the changes in zebrafish aggressive behavior using mirror biting assay [52,56]. In this test, there are two major endpoints that play a key role to measure zebrafish aggressiveness, which are mirror biting time percentage and longest duration in the mirror side. Unchanged mirror biting behavior was observed in C-MNPs-exposed zebrafish exposed to low and high concentrations, which was indicated by the comparable of treated fish average mirror biting time percentage (Figure 3B) and the longest duration in the mirror side time (Figure 3C) to the control group. In addition, although no significant alteration in aggressiveness was observed, the average swimming speed was observed to be significantly lower in the high concentration group (10 ppm) fish and also in the low concentration group (1 ppm) fish, in comparison to the control group (Figure 3A). Supporting this phenomenon, the average of freezing time and rapid movement time ratios demonstrated significant changes in the low and high concentration groups in comparison to the control group (Figure 3D,F). The significant reduction in the rapid movement time ratio (Figure 3F) in both treated groups in comparison to the control group is consistent with the average speed endpoint results. However, the swimming time movement ratio (Figure 3E) showed comparable results in all three groups. The locomotion trajectories of the control, 1 ppm, and 10 ppm C-MNPs-exposed fish in the mirror biting test are summarized in Figure 3G–I and Video S2 for better movement visualization. Overall, these results suggest that no significant alteration was observed in mirror biting aggressiveness tests in zebrafish, even though their locomotor activity was significantly lowered in the zebrafish group exposed to C-MNPs at a high concentration (10 ppm).

### 3.4. C-MNPs Exposure Did Not Change the Predator Avoidance Behavior in Zebrafish

**Figure 4:**
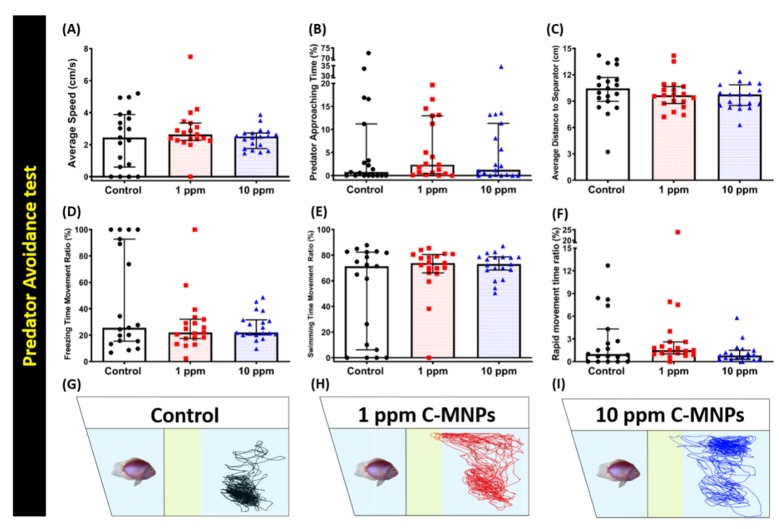
Avoidance behavior endpoint comparisons between control, 1 ppm, and 10 ppm C-MNPs-exposed zebrafish groups after two weeks of exposure. (**A**) Average speed, (**B**) predator approaching time, (**C**) average distance to the separator, (**D**) freezing time movement ratio, (**E**) swimming time movement ratio, and (**F**) rapid movement time ratio were analyzed. The 5 min locomotor trajectories for the control, 1 ppm, and 10 ppm C-MNPs-exposed fish in the predator avoidance test were presented in (**G**–**I**). The data are expressed as the median with interquartile range and were analyzed by the Kruskal-Wallis test with Dunn’s multiple comparisons test as a follow-up test (*n* = 19 for the control group, *n* = 20 for 1 ppm C-MNPs-exposed group, and = 20 for 10 ppm C-MNPs-exposed group).

The avoidance of predators is an innate response for zebrafish, showing unusual freezing and anxious behavior when faced by a natural predator [57,58]. When we exposed the different groups of zebrafish to the predator fish convict cichlid (*Amatitlania nigrofasciata),* we analyzed six different endpoints, namely, average speed, predator approaching time percentage, the average distance to the separator, freezing, swimming, and rapid movement time ratios. In this test, predator approaching time percentage and the average distance to the separator are two major endpoints that play a key role to observe predator avoidance behavior in zebrafish. After the test, these observational endpoints revealed no significant changes in the behavior alteration in predator avoidance test on all of the endpoints of average speed (Figure 4A), predator approaching time (Figure 4B), the average distance to the separator (Figure 4C), freezing time movement ratio (Figure 4D), swimming time movement ratio (Figure 4E) and rapid movement time ratio (Figure 4F), between the control and C-MNPs-exposed zebrafish in the predator avoidance behavior test. The trajectory result issummarized in Figure 4G–I and Video S3 for better movement visualization. These results suggest that C-MNPs exposure does not change the predator avoidance behavior in zebrafish.

### 3.5. C-MNPs Exposure Did Not Alter Shoaling Behavior in the Exposed Zebrafish

**Figure 5:**
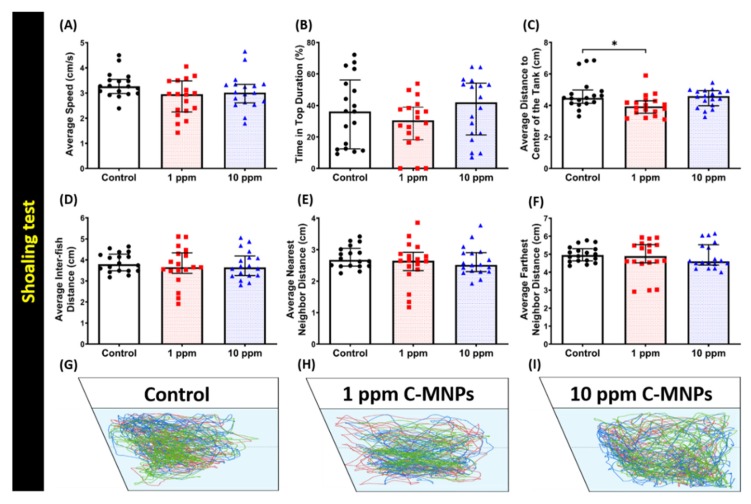
Behavior endpoint comparisons among the control group, 1 ppm, and 10 ppm C-MNPs-exposed zebrafish groups after two weeks of exposure. (**A**) Average speed, (**B**) time in the top duration, (**C**) average distance to the center of the tank, (**D**) average inter-fish distance, (**E**) average nearest neighbor distance, and (**F**) average farthest neighbor distance were analyzed. (**G**–**I**) The 5 min locomotor trajectories for the control, 1 ppm, and 10 ppm C-MNPs-exposed fish in the shoaling test. The data are expressed as the median with interquartile range and were analyzed by the Kruskal-Wallis test, which continued with Dunn’s multiple comparisons test as a follow-up test (*n* = 21 for the control group and 1 ppm MNPs-exposed group, *n* = 24 for 10 ppm C-MNPs, * *p* < 0.05).

Previous studies have established that zebrafish are highly social animals, therefore, they might display dissimilar behavior according to different habitats [56,57,59]. In this experiment, we analyzed the potential social behavior alterations of zebrafish on C-MNPs exposure by conspecific social interaction and shoaling tests. Shoaling or fish swimming together in groups is an innate behavior of avoidance of being captured by predators and reducing anxiety [60,61]. Normally, zebrafish tend to swim together in tight shoal groups when they sense threat [57,62,63]. Six different endpoints, namely, average speed, time on top duration, average shoal area, average inter-fish distance, average nearest neighbor distance, and average farthest neighbor, were analyzed for shoaling test. In this shoaling test, average inter-fish distance, average nearest neighbor distance, and average farthest neighbor distance are the major endpoints to measure the shoals formed by the tested fish.No significant change was observed in the average swimming speed and time in top duration in all the assessed zebrafish groups (Figure 5A,B), although, a significant difference between the low concentration and the control group was seen in average distance to the center of the tank (Figure 5C). In terms of shoal formation, no significant change was observed, which was manifested by no significant changes in the average inter-fish distance, the average nearest neighbor distance, and the farthest neighbor distance (Figure 5D–F). The trajectories of locomotion of zebrafishes in the control, low concentration (1ppm), and high concentration (10 ppm) groups in the shoaling test aresummarized in Figure 5G–I and Video S4 for better movement visualization. The results suggest that C-MNPs exposure does not affect the shoaling behavior in zebrafish.

### 3.6. C-MNPs Exposure Did Not Alter Conspecific Social Interaction Interest in Zebrafish

**Figure 6:**
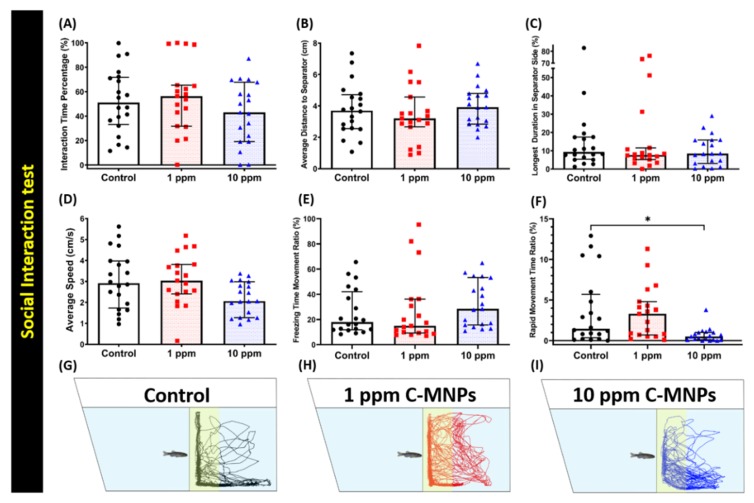
Endpoint comparisons between the control group, 1 ppm, and 10 ppm C-MNPs-exposed zebrafish groups after two weeks of exposure. (**A**) Interaction time percentage, (**B**) average distance to the separator, (**C**) longest duration in the separator side, (**D**) average speed, (**E**) freezing time movement ratio, and (**F**) rapid movement time ratio were analyzed. (**G**–**I**) The 5 min locomotor trajectories for the control, 1 ppm, and 10 ppm C-MNPs-exposed fish in the conspecific social interaction test. The data are expressed as the median with an interquartile range and were analyzed by the Kruskal-Wallis test, which continued with Dunn’s multiple comparisons test as a follow-up test (*n* = 20, * *p* < 0.05).

A specifically designed water tank, with a transparent separator inserted inside the tank, was devised for studying the conspecific social interaction in zebrafish. The behaviors of two separated fish on each side were recorded and compared. All three groups, control, low concentration (1 ppm), and high concentration (10 ppm) were analyzed for six different endpoints after exposure to C-MNPs. Three major endpoints, which are interaction time percentage, the average distance to the separator, and the longest duration in the separator side, are measured to observe whether their social behavior was altered or not. No significant changes were observed in interaction time percentage ratio, the average distance to the separator, longest duration in separator side, average speed, freezing time movement ratio (Figure 6A–E), However, a significant distance between the control and high concentration groups was observed in rapid movement time ratio (Figure 6F). The locomotion trajectories for the control, 1 ppm, and 10 ppm C-MNPs-exposed fish in the conspecific social interaction test are presented in Figure 6G–I and Video S5 for better movement visualization. These results suggest that C-MNPs exposure does not induce substantial changes in the zebrafish behavior at either concentration tested.

### 3.7. Effect of C-MNPs Exposure on Ferric (metal) Content, A Stress Hormone and Oxidative Stress Markers in Brain Tissues

After the behavioral tests, zebrafish were sacrificed and brain tissue samples were prepared, for further analyzing the biochemical marker tests. The ferric (metal) content in the brain tissue was investigated by a colorimetric ferric metal quantification kit; an insignificant change of ferric content in the brain was found in either low (1 ppm) or high (10 ppm) dose groups, respectively, of C-MNPs-exposed zebrafish (Table 1). These results were corroborated by another biomarker of metal-chelating metallothionein, as enzyme-linked immunosorbent assay (ELISA) results showed no significant change was observed in metallothionein content in these fish brains (Table 1). Next, we measure other biomarkers related to reactive oxygen species (ROS) by ELISA to evaluate stress response and anti-oxidative stress capacity. ROS plays a key role in cellular pathways, mediating metal-induced cellular responses, which may further cause damage to proteins leading to apoptosis, or malignant tumors over time [64,65]. We did not find any significant change of ROS level in the brain tissues of C-MNPs-exposed zebrafish, in comparison to the control group (Table 1), suggesting no substantial changes in ROS levels inside the brain. We also measured catalase (CAT), which is established to be an antioxidative response and plays a part in guarding cells from hydrogen peroxide toxicity [66,67,68]. There was no significant change observed in the CAT level between control and C-MNPs-treated groups (Table 1). The thiobarbituric acid reactive substances (TBARS) are formed as a byproduct oflipid peroxidation. It can serve as an indicator of lipid peroxidation caused by oxidative stress in a biological system. A TBARS ELISA kit was used to quantify lipid peroxidation in the samples; which also showed no significant difference in all the three groups (Table 1).

Subsequently, we analyzed cortisol to evaluate the stress level in zebrafish brains after C-MNPs chronic exposure. We did not find any significant changes in the cortisol in any of the C-MNPs-exposed groups in comparison to the control group. To assess if any other changes were taking place inside the brain tissue sample, such as a reduction in oxygen leading to hypoxia or energy reduction, we tested Hif-1α and ATP levels. There was no detectable amount in samples from any of the three groups. In addition, the single-stranded DNA (ssDNA) and lactate dehydrogenase (LDH) tests found no significant change in any of the three study groups, suggesting no DNA and tissue damages in the brain tissues after C-MNPs chronic exposure (Table 1). Together, the above results suggest that C-MNPs does not induce a stress response in exposed zebrafish brain at any of the tested concentrations. Biomarkers related to free radical stress, hypoxia, energy balance, lipid peroxidation, and DNA damage remained unchanged in all the tested groups.

### 3.8. Expression of Neurotransmitters in C-MNPs Exposed Zebrafish Brain

To better understand the behavior alteration mechanism, the neurotransmitters including acetylcholinesterase (AChE), acetylcholine (ACh), serotonin (5-HT), dopamine (DA)and gamma-aminobutyric acid (GABA), in the brain of C-MNPs-exposed zebrafish were investigated by ELISA. AChE has been established to play part in several physiological processes including locomotion and memory by converting ACh to choline and acetate [69,70]. In addition, it is known to impart a swimming disorder in fish when its function been compromised [70,71]. By the ELISA test, no significant change was observed between control and C-MNPs-treated groups for their AChE and ACh levels in the brains. Serotonin is linked to anxiety and depression behavior in the fish [72,73]. Serotonin was found to be comparable between control and C-MNPs-treated groups, supporting an insignificant change in anxiety and depression of zebrafish after exposure to C-MNPs. Dopamine is a neurotransmitter related to the aggressive activity of the brain [74,75]. We found no significant change in the level of dopamine in the zebrafish brain, corroborating results from the behavioral study in aggressiveness test showing no significant change in mirror biting behavior of zebrafish on exposure to C-MNPs. Further, no major changes were observed in the tested groups for gamma-aminobutyric acid (GABA), which regulates the calming effect on brain function [76]. The less variation on neurotransmitter expression between control and C-MNPs-treated groups supporting the idea that waterborne exposure of C-MNPs induces less behavioral alteration compared to their uncoated counterparts [42].

### 3.9. PCA Analysis and Hierarchical Clustering Analysis of Zebrafish Behavioral Endpoints

**Figure 7:**
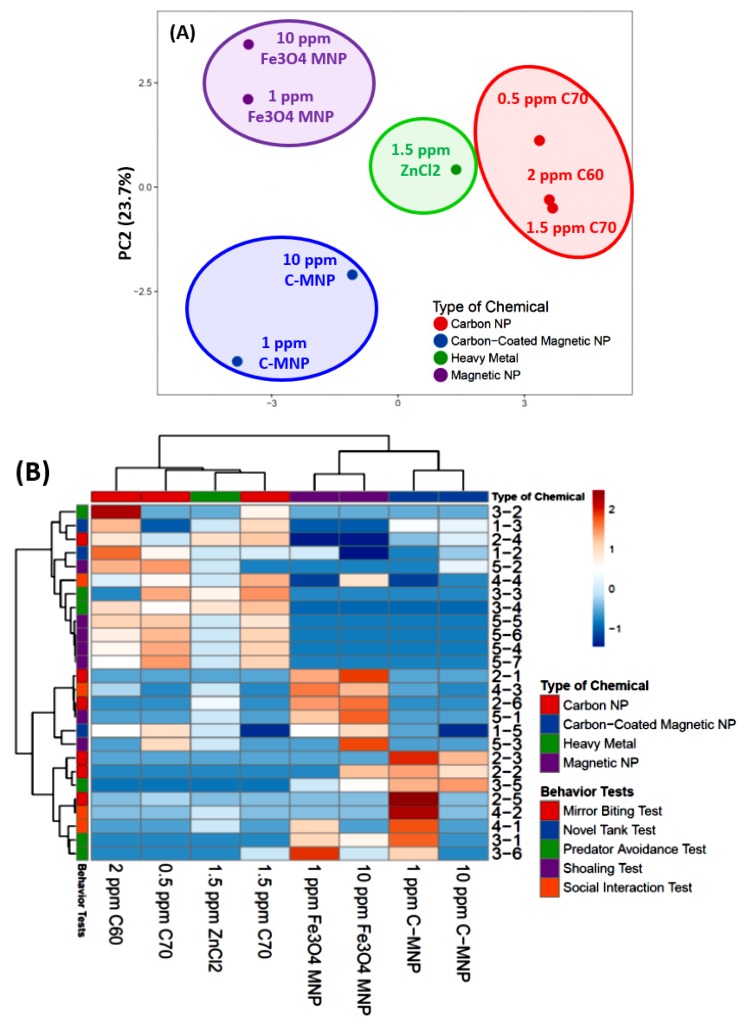
(**A**) Principal Component Analysis (PCA) and (**B**) hierarchical clustering analysis of multiple behavior endpoints in zebrafish after carbon-coated magnetic nanoparticles (C-MNPs) exposure (blue). In addition, the behavioral toxicity data obtained from our previous publications of ZnCl_2_ (green), C60 NPs (red), C70 NPs (red), and uncoated magnetic nanoparticles (purple) chronic exposure in adult zebrafish were also included to compare the behavioral toxicity patterns of each treated group. In Figure 7A, four major groups from each type of chemical were marked with different colors of circles.

Based on five different zebrafish behavioral tests, we are able to explore behavioral phenomics between C-MNPs, uncoated MNPs, and other chemicals by performing the principal component analysis (PCA), hierarchical clustering, and heatmap comparison. Hierarchical clustering revealed the comparability of the zebrafish behavior alteration effect of C-MNPs or other our previously studied chemicals like ZnCl_2_, C60, and C70 fullerenes. Based on PCA grouping, both tested concentrations of C-MNPs (blue color) were categorized in one group while well separated from uncoated MNPs (purple color), ZnCl_2_ (green color), and C60/C70 fullerene (red color) groups (Figure 7A). After performing two-dimensional hierarchical clustering and heatmap generation showing behavior phenomics between MNPs and C-MNPs are close to each other and displaying more distinct with other chemicals like C60/C70 fullerenes or ZnCl_2_ (Figure 7B). In general, for both of the MNPs variables, within the first cluster (C-MNPs, blue color) relatively low behavior endpoint values were reported whereas in the uncoated MNPs (purple color), ZnCl_2_ (green color) and C60/C70 fullerene (red color) variables the first C-MNPs cluster—high behavior endpoint values. Taken together, the degree of similarity between the zebrafish behavior alteration effects of the four different type of chemicals suggests that the low and high doses of C-MNPs produced similar degrees of alteration in all of the zebrafish behavioral parameters while these groups resulted in distinct levels of alteration compared to other chemicals treatment groups like uncoated MNPs, ZnCl_2_, and C60/C70 fullerene.

## 4. Discussion

The toxicity of carbon-coated iron oxide nanoparticles has not been studied widely in model organisms. There is an urgent need for analyzing the toxicity related to C-MNPs because C-MNPs are being considered for use in healthcare such as target drug delivery, photothermal therapy, and theranostics platform. Zebrafish platform provides a useful stage for analysis of behavioral phenotypes as they provide means of high throughput screening tests [77], generating big data for analysis and interpretation for authentication of behavior reliability and safety of the concerned MNPs [78,79]. Aqueous toxicity and neuropharmacology are an emerging field for identifying genes and pathways which can serve as biomarkers or target for drug exposure and generate a big amount of data.

A comprehensive behavioral toxicity approach to evaluate the toxicity of C-MNPs in adult zebrafish has not yet been explored vigorously in the previous studies. The most important achievements of this study are that we have performed a risk assessment of C-MNPs-exposed adult zebrafish based on a panel of behavioral endpoint tests and phenomic analysis to reduce the data complexity. To the best of our knowledge, this is the first time the behavioral toxicity study of C-MNPs is reported. The behavior test panel comprises five major tests including novel tank (test for anxiety), mirror biting (test for aggressiveness), predator avoidance (test for fear), shoaling, and social interaction tests (test for social interaction). The behavior toxicity pattern was assessed on adult zebrafish when chronically exposed to low (1 ppm) and high (10 ppm) concentration of C-MNPs for two weeks, and the results showed that both the low and high concentrations of C-MPNs exposure induced less alterations on behavioral and biochemical markers than their uncoated counterpart in case of adult zebrafish. For behavioral level, we found C-MNPs phenomic analysis outcomes are distinct from those for uncoated MNPs. Our previous publication shows thatwaterborne exposure to uncoated MNPs induce significant elevation for anxiety and reduction for conspecific interaction, shoaling, and memory [42]. However, only slight reduction in locomotion and exploratory behaviors were observed in high dose C-MNPs-treated zebrafish in this study. Overall, almost all of the other behaviors including mirror biting, predator avoidance, shoaling and social interaction, showed no significant alteration in the adult zebrafish exposed to C-MNPs. Furthermore, based on the PCA and heatmap clustering results from the behavior data, C-MNPs showed a distinct pattern thatdistinguishes it from other previously published tested chemicals, including ZnCl_2_ [52], C_60_ NPs [53], C_70_ NPs [80], and uncoated MNPs [42]. This phenomic analysis supports the conclusion that the biosafety of C-MNPs is better than the uncoated MNPs when exposed or delivered to the adult zebrafish.

For the biochemical level, we found C-MNPs exposure do not induce significant changes for all biomarker tested in the brain tissues. On the contrary, our previous publication shows thatwaterborne exposure to uncoated MNPs induce significant elevation for cortisol and AChE, and reduction for serotonin, dopamine, and ACh [42]. Therefore, we provided strong evidence to support the idea that MNPs coated by carbon can strength its magnetic physical property and also do beneficial effects to reduce its potential toxicity when be delivered into animals for the first time [24].

In a study conducted by Kim and colleagues, graphitic carbon coated MNPs (Fe@C NPs) with a diameter of 81 ± 14 nm and 7.0 ± 0.5 nm thickness of carbon layer induced necrotic cell death in human HEK293 cells [81]. The cause of toxicity was graphitic carbon surface encapsulating metalcore. The enhanced cell membrane permeability after exposure to Fe@C NPs and cell cycle arrest contributed sensitization to necrosis. The increase of LDH (a marker for cell damage) was observed in culture media as well. On a similar line, a study conducted by Goya and colleagues demonstrated that carbon encapsulated iron nanoparticles (CEINs) in murine glioma cells (GL261) resulted in high cytotoxic effects in a dose and time-dependent manner [82]. The particle morphology and surface functionalization affect cell cycle progression, particularly in S and G2/M phases. On the contrary, in another study, the metallic iron core nanoparticles coated with carbon did not show any toxic effects on the dendritic cell viability [83]. The magnetic signal generated demonstrated the internalization of nanoparticles in a size range of 10–200 nm. The safety and efficacy of colloidal Fe_3_O_4_@C were also proposed by a study when these nanoparticles were exposed to human breast adenocarcinoma MCF-7 cell line [11]. All these findings on the cell lines, therefore, propose contradictory results, which need more substantial data to confirm the safety of carbon-coated magnetic nanoparticles.

Compared to cell level studies, there is a shortage of studies on C-MNPs effect on living organisms. Herrmann and colleagues injected carbon encapsulated iron carbide nanoparticles intravenously to C57BL/6 mice and found carbon encapsulated MNPs were found to localize predominantly in the reticuloendothelial system (like the lung and liver), proposed long term exposure did not result in any injury or tissue damage the animals [84]. In agreement with previous findings reported in rodents, the present behavioral study found that two weeks of C-MNPs exposure did not cause a significant change in zebrafish behaviors and biochemical marker expression, supporting its in vivo compatibility and high biosafety. We proposed the possible mechanism for less toxicity of C-MNPs than uncoated MNPs might be contributed from the protective effect of carbon coating on preventing corrosion and oxidation for the internal MNPs core, since Fe_3_O_4_ MNPs in a small size (5–15 nm) has been reported with chemical corrosion instability, which may cause partial oxidation into γ-Fe_2_O_3_ [14,15].

## 5. Conclusions

Herein, we have compiled a detailed and comprehensive synopsis of behavior and biochemical changes happening in the zebrafish brain upon exposure to low (1 ppm) and high (10 ppm) concentration of C-MNPs. Less toxic effect than uncoated MNPs was observed at both behavioral and biochemical levels in adult zebrafish in either low or high doses examined. Our results suggest that exposure of C-MNPs is safer in comparison to uncoated MNPs for which we have published data in our earlier publication [42]. Hence more studies on different parameters of long term exposure, dosage, and environmental condition are required to assess the toxicity and safety for collecting big data to prepare a further course of action for the usage of C-MNPs. Carbon-coated MNPs have not caused considerable abnormalities to the zebrafish at behavior and biochemical levels in the current work. However, a reduction in locomotion and exploratory behavior at the high dose of the C-MNPs group still can be detected and cannot be neglected. Further development of a new generation of the surface-modified version of MNPs is considered necessary to improve its biosafety and biocompatibility. In the future, this phenomic approach can also be applied to design research work to perform toxicity or biosafety assay in a model organism in relation to different sizes or different decorated MNPs. This is a first in its kind ofstudy related to the toxicity effect of C-MNPs which still awaits data from different perspectives to provide appropriate safety guidelines for clinical usage.

## Figures and Tables

**Table 1:** Comparison of biomarker expression in the brain for carbon-coated magnetic nanoparticles (MNPs) exposed zebrafish. Data are expressed as the mean ± SEM. ns = not significant and tested by one-way ANOVA.

Biomarker	WT	C-MNP		C-MNP		Unit	Significance	ANOVA	*p* Value
		(1 ppm)		(10 ppm)				*F* Value	
Ferric ion	1.070 ± 0.4689	1.821 ± 0.652	^ns^	0.9054 ± 1.025	^ns^	pg/ug of total protein	NO	F (2,6) = 1.089	*p* = 0.3949
Metallothionein	34.41 ± 9.015	40.69 ± 14.05	^ns^	27.81 ± 3.921	^ns^	pg/ug of total protein	NO	F (2,6) = 0.423	*p* = 0.6728
ROS	317.5 ± 70.68	428.8 ± 98.14	^ns^	223.6 ± 9.179	^ns^	U/ug of total protein	NO	F (2,6) = 2.152	*p* = 0.1975
CAT	29.05 ± 7.592	42.22 ± 10.61	^ns^	23.05 ± 1.903	^ns^	U/ug of total protein	NO	F (2,6) = 1.658	*p* = 0.2672
TBARS	183.4 ± 52.11	183.9 ± 37.91	^ns^	97.49 ± 6.354	^ns^	ng/ug of total protein	NO	F (2,6) = 1.770	*p* = 0.2488
Cortisol	290.6 ± 80.02	260.7 ± 85.90	^ns^	142.3 ± 10.09	^ns^	pg/ug of total protein	NO	F (2,6) = 1.330	*p* = 0.3326
Hif-1α	147.7 ± 40.59	200.5 ± 60.90	^ns^	109.4 ± 4.572	^ns^	pg/ug of total protein	NO	F (2,6) =1.167	*p* = 0.3732
ATP	3074 ± 933.2	3896 ± 1235	^ns^	1999 ± 152.5	^ns^	pg/ug of total protein	NO	F (2,6) = 1.123	*p* = 0.3852
Hif-1α	147.7 ± 40.59	200.5 ± 60.90	^ns^	109.4 ± 4.572	^ns^	pg/ug of total protein	NO	F (2,6) =1.167	*p* = 0.3732
LDH	24.93 ± 6.257	35.61 ± 9.463	^ns^	18.76 ± 1.228	^ns^	ng/ug of total protein	NO	F (2,6) = 1.673	*p* = 0.2646
Acetylcholine esterase	50.59 ± 15.76	86.45 ± 29.01	^ns^	55.37 ± 2.924	^ns^	U/ug of total protein	NO	F (2,6) = 1.035	*p* = 0.4109
Acetylcholine	242.9 ± 58.99	414.5 ± 131.4	^ns^	221.6 ± 12.99	^ns^	U/ug of total protein	NO	F (2,6) = 1.605	*p* = 0.2764
5-HT	6.242 ± 1.861	8.573 ± 2.885	^ns^	4.663 ± 0.1496	^ns^	ng/ug of total protein	NO	F (2,6) = 0.983	*p* = 0.4273
Dopamine	377.3 ± 88.96	521.1 ± 186.3	^ns^	297.7 ± 5.453	^ns^	pg/ug of total protein	NO	F (2,6) = 0.901	*p* = 0.4545
GABA	1.747 ± 0.4157	2.688 ± 0.7480	^ns^	1.491 ± 0.0231	^ns^	U/ug of total protein	NO	F (2,6) = 1.402	*p* = 0.3165

## Supplementary Materials

The following are available online at https://www.mdpi.com/1420-3049/25/9/2256/s1, Table S1: Summary of behavior endpoints measured in this study, Video S1: Locomotion of wild type and C-MNPs exposed zebrafish in the novel tank test, Video S2: Locomotion of wild type and C-MNPs exposed zebrafish in the mirror biting test, Video S3: Locomotion of wild type and C-MNPs exposed zebrafish in the predator avoidance test, Video S4: Locomotion of wild type and C-MNPs exposed zebrafish in the social interaction test, Video S5: Locomotion of wild type and C-MNPs exposed zebrafish in the shoaling test. All of the videos were played at 10x speed, except Video S1 (5x speed).

## Appendix A

**Table A1:** Summary of behavior endpoints measured in this study.

Zebrafish Behavior Endpoints Legends		
**1**	**Novel Tank Exploration Test**	
	1-1	Average Speed
	1-2	Freezing Time Movement Ratio
	1-3	Time in Top Duration
	1-4	Number of Entries to The Top
	1-5	Latency to Enter The Top
	1-6	Total Distance Traveled in The Top
**2**	**Mirror Biting Test**	
	2-1	Average Speed
	2-2	Mirror Biting Time Percentage
	2-3	Longest Duration in The Mirror Side
	2-4	Freezing Time Movement Ratio
	2-5	Swimming Time Movement Ratio
	2-6	Rapid Movement Time Ratio
**3**	**Predator Avoidance Test**	
	3-1	Average Speed
	3-2	Predator Approaching Time
	3-3	Average Distance to Separator
	3-4	Freezing Time Movement Ratio
	3-5	Swimming Time Movement Ratio
	3-6	Rapid Movement Time Ratio
**4**	**Social Interaction Test**	
	4-1	Interaction Time Percentage
	4-2	Longest Duration in Separator Side
	4-3	Average Speed
	4-4	Average Distance to Separator
**5**	**Shoaling Test**	
	5-1	Average Speed
	5-2	Time in Top Duration
	5-3	Average Distance to Center of the Tank
	5-4	Average Inter-fish Distance
	5-5	Average Shoal Area
	5-6	Average Nearest Neighbor Distance
	5-7	Average Farthest Neighbor Distance
